# Diabetic retinopathy at the Yaoundé Central Hospital in Cameroon: epidemiology and angiographic findings

**Published:** 2012-11-16

**Authors:** Chantal Nanfack, Godefroy Koki, Lawrence Mbuagbaw, Assumpta Lucienne Bella

**Affiliations:** 1 Department of Ophthalmology, Faculty of Medicine and Biomedical Sciences, University of Yaounde 1, PO Box 1364,Yaounde, Cameroon; 2 Hôpital militaire de Région N 1, Cameroon; 3 Centre for the Development of Best Practices in Health (CDBPH), Yaounde Central Hospital, Henri Dunant Avenue, Messa, PO Box 87, Yaounde, Cameroon; 4 Yaounde Central Hospital, Henri Dunant Avenue, Messa, PO Box 87, Yaounde, Cameroon

**Keywords:** Diabetes, diabetic retinopathy, angiography, hypertension

## Abstract

We carried out a cross-sectional analytical survey using data from patients who had done Fluorescein Angiography at the Yaounde Central Hospital Diabetic Retinopathy Prevention and Management Project between October 2007 and January 2010 to identify the risk factors, incidence and severity of different types of diabetic retinopathy. Data from 239 males (57.0%) and 180 females (43.0%) with diabetic retinopathy were included. Their mean age was 58.2 years. A majority of them were living with type II diabetes (96.2%). The mean duration of diabetes was 8.2 years. About sixty percent had both diabetes and hypertension. The average level of glycated haemoglobin was 9.72% (range 6-17.7%). Amongst the 419 patients with diabetic retinopathy, 292(69.7%) had non-proliferative diabetic retinopathy. One hundred and twelve (26.7%) of those with proliferative diabetic retinopathy had a formal indication for laser photocoagulation. Fifteen patients (3.6%) presented with complicated forms of proliferative diabetic retinopathy. Diabetic maculopathy was present in 30 patients (7.2%). Diabetic retinopathy is a frequent complication of diabetes in our setting which stems from inadequate emphasis on preventive measures. The technical requirements for managing some of the existing complications are still unavailable. Fluorescein Angiography is an important diagnostic tool which should be popularized.

## To the editors of the Pan African Medical Journal

Diabetic retinopathy (DR) is one of many complications of diabetes. It is the fifth cause of blindness worldwide and the first cause of blindness before the age of 50 in developed countries [1, 2]. It is responsible for close to 5% of all cases of blindness [2]. The incidence and prevalence of DR are proportional to the duration of diabetes and life expectancy. Hence, after 20 years living with diabetes, 90% of those with type I and 60% of those with type II develop DR [3, 4]. In Cameroon, early screening, diagnosis and timely management of ocular disease is poor and reflected by the burden of visual impairment (4.6% increase in the past decade) despite the preventable nature of this affliction [5, 6].

In order to report the risk factors, incidence and severity of different types of DR we conducted a cross-sectional analytical study in patients who had done Fluorescein Angiography (FA)at the Yaounde Central Hospital (YCH) Diabetic Retinopathy Prevention and Management Project (DRPMP). We obtained ethical clearance from the YCH Ethics Board and consulted patient records from October 2007 to January2010. We included all patients who were diagnosed with DR using FA.DR was classified using the French Association for the Study of Diabetes and Metabolic Diseases (ALFEDIAM) classification [7].

**Figure 1:**
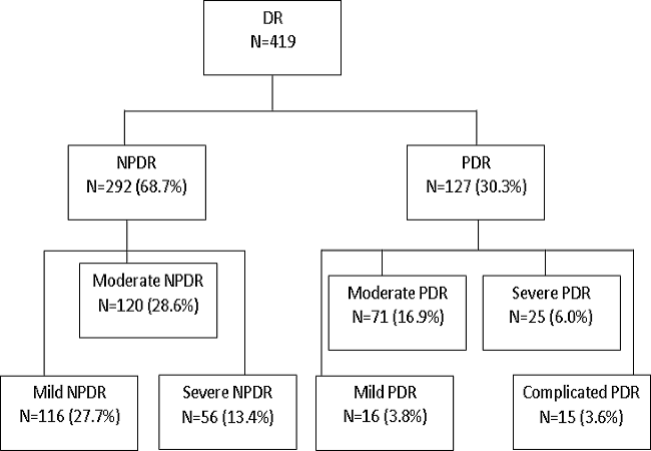
Distribution of participants by severity of diabetic retinopathy; DR: Diabetic Retinopathy; NPDR: Non-proliferative DR; PDR: Proliferative DR

Data were collected from 419 eligible patient files belonging to 239 males (57.0%) and 180 females (43.0%) who had DR.The incidence of DR was39.9%.Their average age was 58.2 years (range 29-87). The average age for patients with type I and type II diabetes was 51.6 and 59.0 years respectively. Most had type II diabetes (96.2%). The mean duration of diabetes before the diagnosis of DR was 8.2 years.Two hundred and fifty-two(60.1%) had both diabetes and high blood pressure (HBP). The average level of glycated haemoglobin was 9.7%(range 6-17.7%). Thirty patients (7.15%) had diabetic maculopathy; 27(6.43%) with maculao edema and 3(0.7%) had ischemic maculopathy.Amongst the patients with proliferative DR, 26.7%(112) had a formal indication for laser photo coagulation. Fifteen patients (3.6%) presented with complicated forms of proliferative DR.However,non-proliferative DR was more frequent (68.7%) (Figure 1).

The mean duration of diabetes before diagnosis of DR is comparable with other reports [8]. Glycaemic control was poor(9.72%).Strict control of glycaemia is necessary for the prevention of DR [9]. Concomitant HBP and diabetes were not a risk factor for DR in this study.

Poor knowledge about the complications of diabetes, poor compliance with treatment and the absence of comprehensive health insurance may explain these high figures. Many of these patients suffered from complicated forms of retinal disease that require currently unavailable endocular surgery. Additional efforts in primary prevention through lifestyle modifications and risk factor prevention are needed. Secondary prevention by better glycaemic control and early screening for DR should be implemented.

## Conclusion

DR is frequent in our setting. Even though it occurs earlier in patients with type I diabetes, it is more frequent in patients with type II diabetes. A yearly eye exam in diabetics is highly recommended as such preventive measures are cost-effective [8, 10]. FA is a useful tool for typing DR.
